# Feststellung des irreversiblen Hirnfunktionsausfalls in Deutschland – Umsetzung der Richtlinie der Bundesärztekammer

**DOI:** 10.1007/s00115-023-01520-5

**Published:** 2023-07-18

**Authors:** Olaf Hoffmann, Farid Salih, Florian Masuhr

**Affiliations:** 1 Klinik für Neurologie, Alexianer St. Josefs-Krankenhaus Potsdam, Potsdam, Deutschland; 2 Medizinische Hochschule Brandenburg Theodor Fontane, Neuruppin, Deutschland; 3 Neurocure Clinical Research Center, Charité-Universitätsmedizin Berlin, Berlin, Deutschland; 4 Klinik für Neurologie, Charité-Universitätsmedizin Berlin, Berlin, Deutschland; 5 Klinik für Neurologie, Bundeswehrkrankenhaus Berlin, Berlin, Deutschland; 6 Klinik für Neurologie, Alexianer St. Josefs-Krankenhaus Potsdam, Allee nach Sanssouci 7, 14471 Potsdam, Deutschland

**Keywords:** Hirntod, Organspende, Organtransplantation, CT-Angiographie, Apparative Zusatzdiagnostik, Brain death, Organ donation, Organ transplantation, CT angiography, Ancillary studies

## Abstract

**Hintergrund:**

Seit Inkrafttreten der 4. Fortschreibung der Richtlinie der Bundesärztekammer gelten in Deutschland wesentliche neue Normierungen in der Diagnostik des irreversiblen Hirnfunktionsausfalls (IHA). Hierzu zählen die Qualifikationsanforderungen an die Untersucher, zugelassene Verfahren zur apparativen Zusatzdiagnostik und eine Präzisierung zur Abfolge der Prozessschritte.

**Fragestellung:**

Untersuchung der Auswirkungen auf die Praxis der IHA-Feststellung.

**Methodik:**

Deskriptive Auswertung der Dokumentation der Deutschen Stiftung Organtransplantation über IHA-Diagnostik im Vergleich der Zeiträume Juli 2011 bis Juni 2015 (3. Fortschreibung) und Juli 2015 bis Juni 2019 (4. Fortschreibung).

**Ergebnisse:**

Die Zahl der erfassten Patienten sank von 6100 auf 5403. Die stärkste Abnahme betraf Krankenhäuser ohne Neurochirurgie. Kinder unter 14 Jahren waren nicht betroffen. Die klinische Diagnostik erfolgte ab Juli 2015 vermehrt im Bereitschaftsdienst durch externe neurologische Konsiliare. Zusatzdiagnostik erhielten nun 83,8 % der Patienten, zuvor 80,1 %. Die neu etablierte CTA wurde bei 23,2 % eingesetzt. Sie wies in 89,4 % den zerebralen Zirkulationsstillstand nach. Die Zeitdauer zwischen erstmaliger Feststellung der klinischen Ausfallzeichen und Feststellung des IHA stieg von 7,0 ± 12,7 h auf 8,2 ± 14,2 h. Der IHA wurde mit 95,3 % gegenüber 96,6 % geringfügig seltener festgestellt.

**Diskussion:**

Die neuen Normierungen wurden richtlinienkonform umgesetzt. Der Bedarf an konsiliarischer Unterstützung durch Neurologen und Neurochirurgen sowie der Zeitbedarf für die IHA-Feststellung haben zugenommen. Negative Effekte auf die pädiatrische IHA-Diagnostik wurden nicht deutlich. Die CTA wird bei Erwachsenen als neues zusatzdiagnostisches Verfahren flächendeckend erfolgreich eingesetzt.

## Abstract (en)

**Background:**

The 4th update of the guidelines of the German Medical Association on the diagnosis of irreversible loss of brain function (brain death, BD) has introduced important new regulations regarding the required qualification of the examiners, approved procedures for ancillary testing, and a clarification regarding the sequencing of diagnostic steps.

**Objective:**

Investigation of the implementation and practical effects on the diagnosis of brain death.

**Method:**

Descriptive evaluation of the routine documentation of the German Organ Procurement Organization, comparing the periods July 2011–June 2015 (3rd update) and July 2015–June 2019 (4th update).

**Results:**

Patient numbers decreased from 6100 to 5403. The largest decrease affected hospitals without neurosurgery. Children were not affected. With the 4th update, clinical diagnostics were increasingly performed during on-call hours by external neurologists. Of the patients 83.8% now received ancillary tests compared to 80.1% previously. Computed tomography angiography (CTA), first introduced in the 4th update, was applied in 23.2% and established complete loss of cerebral circulation in 89.4%. The time between first documentation of the clinical signs of BD and certification of BD increased from 7.0 ± 12.7 h to 8.2 ± 14.2 h. The diagnosis was slightly less frequent with 95.3% compared to 96.6%.

**Conclusion:**

The updated standards were implemented in accordance with the guidelines. The demand for external consulting neurologists and neurosurgeons as well as the time required for BD assessment have increased. Negative effects on pediatric BD diagnostics were not apparent. CTA is widely and successfully used in adults as a new ancillary diagnostic procedure.

## Hintergrund

Die Feststellung des irreversiblen Ausfalls der Gesamtfunktion des Großhirns, des Kleinhirns und des Hirnstamms (IHA) ist in Deutschland durch die Richtlinie der Bundesärztekammer normiert. In der 5. Fortschreibung (FS) vom September 2022 [3] wurden im Rahmen einer turnusmäßigen Überprüfung Hinweise zum Apnoetest unter extrakorporaler Zirkulation sowie zur digitalen Elektroenzephalographie (EEG) aufgenommen und die Befähigung von Fachärzten für Kinder- und Jugendchirurgie für die pädiatrische IHA-Diagnostik ergänzt.

Stärkere Änderungen gegenüber den früheren Regelungen traten mit der 4. FS im Juli 2015 in Kraft [2], darunter:

- An der klinischen Diagnostik dürfen nur Fachärzte mit mehrjähriger Erfahrung in der Intensivbehandlung von Patienten mit akuten schweren Hirnschädigungen mitwirken.
- Mindestens einer der klinischen Untersucher muss Facharzt für Neurologie oder Neurochirurgie sein oder (bei Patienten bis zum Beginn des 15. Lebensjahrs) die Zusatzbezeichnung Neuropädiatrie führen.
- Vor Beginn des 15. Lebensjahrs muss ein intensivmedizinisch weitergebildeter Facharzt für Kinder- und Jugendmedizin beteiligt sein.
- Zum Nachweis des zerebralen Perfusionsstillstandes ist die CT-Angiographie (CTA) nach definiertem Protokoll zulässig.

Die Krankenhäuser stehen somit vor veränderten logistischen Herausforderungen, qualifizierte Untersucher und diagnostische Verfahren zur IHA-Feststellung zeitgerecht zur Verfügung zu stellen [1, 7]. Die vorliegende Arbeit untersucht Änderungen in der Praxis der IHA-Feststellung nach Inkrafttreten der 4. FS.

## Material und Methoden

Die Deutsche Stiftung Organtransplantation (DSO) erfasst als koordinierende Stelle gemäß Transplantationsgesetz (TPG) gemeldete Patienten, die im Krankenhaus infolge einer akuten Hirnschädigung versterben. Dies umfasst auch Verstorbene, bei denen der IHA nicht festgestellt wurde oder keine Organspende erfolgte. Die Daten wurden den Autoren von der DSO zur Verfügung gestellt. Sie wurden zuvor vollständig anonymisiert, um eine Zuordnung zu Personen, Krankenhäusern oder Regionen auszuschließen. Verglichen wurden die Zeiträume Juli 2011 bis Juni 2015 (3. FS) bzw. Juli 2015 bis Juni 2019 (4. FS). Als Kategorien dienten

- die Altersgruppen laut Richtlinie (Neugeborene und Säuglinge vor Beginn des 3. Lebensjahres, Kinder ab Beginn des 3. Lebensjahres, Erwachsene und Jugendliche ab Beginn des 15. Lebensjahres),
- das Vorliegen einer primären oder sekundären Hirnschädigung als Ursache des IHA,
- die Ätiologie des IHA (spontane intrakranielle Blutung [ICB], Schädel-Hirn-Trauma [SHT], Hirninfarkt, Hirntumor, ZNS-Infektion, andere),
- die Art des Krankenhauses (A-Universitätskliniken, B‑Krankenhäuser mit Neurochirurgie, C‑Krankenhäuser ohne Neurochirurgie),
- Zugehörigkeit zu einer von sieben (anonymisierten) DSO-Verwaltungsregionen.

Patientenbezogen erfasst wurden Zahl, Zeitpunkt und Ergebnis der klinischen Untersuchungen und der apparativen Zusatzdiagnostik (ZD) zur IHA-Feststellung. Anhand des zeitlichen Ablaufes erfolgte eine Zuordnung zu Untersuchungsgängen. Unterschieden wurde zwischen Kernarbeitszeit (werktags 08:00–17:00) und Bereitschaftsdienst (außerhalb davon; BD). Bei den Untersuchern wurde die Fachrichtung erfasst und zwischen Ärzten des Krankenhauses und externen Konsilen unterschieden. Schließlich wurden das Endergebnis der Diagnostik sowie eine etwaige Organspende erfasst.

Die Auswertung erfolgte deskriptiv mittels Microsoft Access und Microsoft Excel (Microsoft, Redmond, CA, USA) sowie GraphPad Prism (GraphPad Software, Boston, MA, USA). Vorab wurden keine Hypothesen gebildet. Berichtet werden Anzahl, Mittelwert ± Standardabweichung und Prozentanteile. Die zeitabhängige Wahrscheinlichkeit eines negativen CTA-Ergebnisses wurde mittels Kaplan-Meier-Analyse dargestellt.

## Ergebnisse

### Patienten

**Figure 1:**
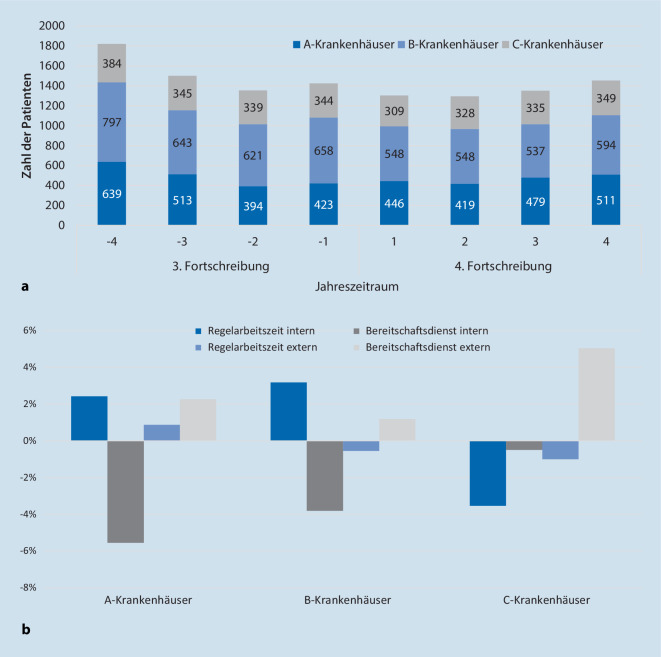

Von Juli 2011 bis Juni 2019 wurde bei 11.503 Patienten eine IHA-Diagnostik begonnen (Tab. 1). Es erfolgten 28.388 klinische Untersuchungen und 10.434 apparative ZD. Der IHA wurde bei 11.093 Patienten festgestellt; bei etwa zwei Dritteln erfolgte eine Organspende. Die jährliche Fallzahlentwicklung ist in Abb. 1a dargestellt. Seit Inkrafttreten der 4. FS erfolgten jährlich 1351 ± 73 Untersuchungen; zuvor 1525 ± 206. Die Fallzahl sank in den Universitätskliniken um 5,8 %, in den B‑Krankenhäusern um 18,1 % und in den C‑Krankenhäusern um 6,4 %.

**Table 1:** 

		3. Fortschreibung	4. Fortschreibung
Patienten	n	6100	5403
	*n*/Jahr (m ± SD)	1525 ± 206	1351 ± 73
	Alter (m ± SD)	54,1 ± 18,1	54,2 ± 18,9
	< 2 Jahre	48 (0,8 %)	59 (1,1 %)
	2–13 Jahre	102 (1,7 %)	129 (2,4 %)
	> 13 Jahre	5950 (97,5 %)	5215 (96,5 %)
Erkrankungen	Primäre Hirnschädigung	4896 (80,3 %)	4044 (74,8 %)
	Spontane ICB	3016 (49,4 %)	2601 (48,1 %)
	SHT	959 (15,7 %)	734 (13,6 %)
	Hirninfarkt	682 (11,2 %)	512 (9,5 %)
	Hirntumor	33 (0,5 %)	31 (0,6 %)
	ZNS-Infektion	53 (0,9 %)	56 (1,0 %)
	Andere	153 (2,5 %)	110 (2,0 %)
	Sekundäre Hirnschädigung	1204 (19,7 %)	1359 (25,2 %)
Einrichtungen	A – Universitätsklinika	1969 (32,3 %)	1855 (34,31 %)
	B – Andere, mit NCH	2719 (44,6 %)	2227 (41,2 %)
	C – Andere, ohne NCH	1412 (23,1 %)	1321 (24,4 %)
Diagnostik	Klinische Untersuchungen (*n*)	15.254	13.134
	Untersuchungsgänge je Patient (m + SD)	1,3 ± 0,5	1,2 ± 0,5
	*n* = 1	4330	4179
	*n* = 2	1703	1166
	*n* = 3	59	53
	*n* = 4	7	5
	*n* = 5	1	–
	Patienten mit ZD	4885 (80,1 %)	4530 (83,8 %)
	EEG (*n* Untersuchungen/*n* Patienten)	3150/2912 (47,7 %)	2198/2115 (39,1 %)
	FAEP	62/60 (1,0 %)	36/36 (0,7 %)
	SEP	117/112 (1,8 %)	64/63 (1,2 %)
	Sonographie	1692/1553 (25,5 %)	1141/1064 (19,7 %)
	Szintigraphie	456/450 (7,4 %)	189/189 (3,5 %)
	CTA	–	1277/1253 (23,2 %)
	DSA	42/42 (0,7 %)	8/8 (0,1 %)
Klinische Untersucher	Neurologie	5073 (33,3 %)	5432 (41,4 %)
	Anästhesie	4539 (29,8 %)	3152 (24,0 %)
	Neurochirurgie	2370 (15,5 %)	2606 (19,8 %)
	Innere Medizin	902 (5,9 %)	659 (5,0 %)
	Pädiatrie	225 (1,5 %)	220 (1,7 %)
	Chirurgie	91 (0,6 %)	63 (0,5 %)
	Neuropädiatrie	29 (0,2 %)	101 (0,8 %)
	Radiologie	8 (0,1 %)	3 (0 %)
	Unbekannt	2017 (13,2 %)	898 (6,8 %)
Neuromediziner an allen klinischen Untersuchungen beteiligt	Ja	4527 (74,2 %)	5153 (95,4 %)
	Nein	920 (15,1 %)	86 (1,6 %)
	Unbekannt	653 (10,7 %)	164 (3,0 %)
Neuromediziner an der letzten klinischen Untersuchungen beteiligt	Ja	4836 (79,3 %)	5207 (96,4 %)
	Nein	616 (10,1 %)	44 (0,8 %)^a^
	Unbekannt	648 (10,6 %)	152 (2,8 %)
Ergebnisse	IHA festgestellt	5894 (96,6 %)	5149 (95,3 %)
	– Organspende	3860 (63,3 %)	3449 (63,8 %)
	– Ablehnung	1734 (28,4 %)	1332 (24,7 %)
	– Medizinische Kontraindikation	254 (4,2 %)	296 (5,5 %)
	– Kreislaufstillstand	24 (0,4 %)	20 (0,4 %)
	– Nicht erfasst	22 (0,4 %)	52 (1,0 %)
	IHA nicht festgestellt	206 (3,4 %)	254 (4,7 %)
	– Voraussetzungen nicht erfüllt	4 (0,1 %)	9 (0,2 %)
	– Kein Hirnfunktionsausfall	38 (0,6 %)	60 (1,1 %)
	– Therapiereduktion	105 (1,7 %)	130 (2,4 %)
	– Kreislaufstillstand	59 (1,0 %)	55 (1,0 %)

*m±SD* Mittelwert±Standardabweichung, *CTA* CT-Angiographie, *DSA* digitale Subtraktionsangiographie, *EEG* Elektroenzephalographie, *FAEP* frühe akustisch evozierte Potentiale, *ICB* intrazerebrale Blutung, *SEP* Somatosensibel evozierte Potentiale, *SHT* Schädel-Hirn-Trauma, *NCH* Neurochirurgie, *ZD* Zusatzdiagnostik

^a^kein Fall abgeschlossen

Zum IHA führten überwiegend primäre Hirnschädigungen. Der Anteil sekundärer Hirnschädigungen war im Gesamtzeitraum in den C‑Krankenhäusern mit 44,0 % deutlich höher als in den A‑ und B‑Krankenhäusern (17,5 % bzw. 14,0 %). Er stieg im Zeitverlauf an, am stärksten in den C‑Krankenhäusern mit 47,4 % (4. FS) gegenüber 40,8 % (3. FS).

Der Anteil von Patienten mit Diagnose des IHA sank nach Inkrafttreten der 4. FS in den Universitätskliniken von 96 auf 95,1 %, in den B‑Krankenhäusern von 97 auf 96,0 % und etwas deutlicher in den C‑Krankenhäusern von 95 auf 92,7 %. Die Zeitspanne zwischen erstmaliger Feststellung der klinischen Ausfallzeichen und Diagnose des IHA verlängerte sich von 7,0 ± 12,7 h auf 8,2 ± 14,2 h.

### Klinische Diagnostik

Klinische IHA-Diagnostik erfolgte überwiegend in der Regelarbeitszeit. Dies war mit 59,2 % bzw. 64,8 % der Untersuchungen in den A‑ und B‑Krankenhäusern häufiger als in den C‑Krankenhäusern (47,3 %). Externe Konsile (10,4 % der Untersuchungen) erfolgten nahezu ausschließlich durch Fachärzte für Neurologie (93,3 %) und Neurochirurgie (6,4 %). Nach der Novelle erfolgen 5 externe Konsile durch Pädiater. A‑ und B‑Krankenhäuser nutzten externe Konsile selten, sowohl im Geltungszeitraum der 3. FS (4,1 % bzw. 7,3 %) als auch der 4. FS (7,3 % bzw. 6,2 %). In den C‑Krankenhäusern war der Anteil mit 24,4 % primär höher und nahm mit der 4. FS auf 28,5 % weiter zu. Externe Konsile erfolgten meist im BD (3. FS, 67,1 %; 4. FS, 74,9 %).

### Apparative Zusatzdiagnostik

Mindestens eine ZD erhielten 81,8 % der Patienten (Tab. 1). Nach der Novelle nahm dieser Anteil geringfügig zu. Hierbei trat die neu etablierte CTA mit 26,0 % an die zweite Stelle der ZD. Dagegen nahm die Nutzung anderer ZD ab, am stärksten die des EEG. ZD wurde in A‑ und B‑Krankenhäusern meist selbständig durchgeführt (90,4 % bzw. 80,7 %), in C‑Krankenhäusern überwiegend von externen Konsiliaren (54,8 %). Selbständige ZD erfolgte meist während der Kernarbeitszeit (74,4 %), konsiliarische meist im BD (73,6 %). Nach Inkrafttreten der 4. FS stieg die selbständige ZD in den B‑ und C‑Krankenhäusern um je 5,8 % an, v. a. durch Einsatz der CTA.

### Nutzung der CT-Angiographie

**Figure 2:**
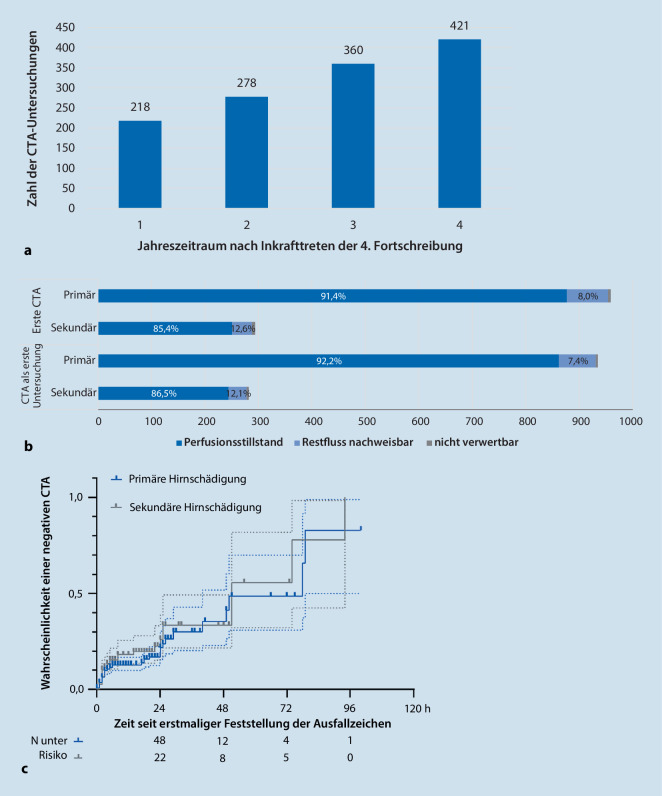

Im Geltungszeitraum der 4. FS erfolgten 1277 CTA bei 1253 Patienten (Abb. 2a). Erhaltene Perfusion fand sich häufiger bei sekundärer als bei primärer Hirnschädigung (Abb. 2b). Mit zunehmender Dauer seit Feststellung der klinischen Ausfallzeichen sank der Anteil mit nachgewiesenem Perfusionsstillstand (Abb. 2c). Als nicht verwertbar wurden 0,9 % der CTA gemeldet; zu den Ursachen liegen keine Informationen vor.

Bei 1217 Patienten erfolgte die CTA als erste ZD. Restperfusion zeigten 103 Patienten (8,5 %). Hiervon erhielten 17 später erneut eine CTA. Positiv waren 7 dieser CTA, während 10 weiterhin Perfusion zeigten. Tendenziell häufiger wurden in dieser Situation positive Befunde mittels EEG (22 von 27) oder Szintigraphie (3 von 3) erhoben, dagegen nur bei 4 von 8 transkraniellen Doppler- bzw. Duplexsonographien (TCD).

Die CTA erfolgte bei 70 klinisch nicht vollständig beurteilbaren Patienten, um die Vollständigkeit des IHA zu belegen. Bei diesen waren 56 CTA positiv, während 14 Perfusion aufwiesen. Dieser Anteil war mit 20,0 % höher als bei den vollständig beurteilbaren Patienten mit klinischem Hirnfunktionsausfall (8,5 %).

Im TCD wurde mit 14,2 % von 2753 auswertbaren Untersuchungen häufiger Perfusion beschrieben als in der CTA (9,9 % von 1266) sowohl bei primärer (13,0 % vs. 8,7 %) als auch bei sekundärer Hirnschädigung (17,1 % vs. 13,7 %). Lediglich 11 Patienten erhielten TCD und CTA im selben Untersuchungsgang. Wo diskrepante Ergebnisse dokumentiert wurden, belegte die jeweils spätere ZD (4 CTA, 3 TCD) den Perfusionsstillstand.

### Abfolge der Prozessschritte und diagnostisches Vorgehen

Die unabhängige Feststellung der klinischen Ausfallzeichen durch zwei hierfür qualifizierte Ärzte erfolgte vor Inkrafttreten der 4. FS bei 5748 von 7705 Untersuchungsgängen zeitgleich (74,6 %) oder im Abstand von weniger als 1 h (*n* = 1426; 18,5 %). Danach betraf dies 5652 (85,1 %) bzw. 867 (13,1 %) von 6638 Untersuchungen. Durchschnittlich verkürzte sich der Abstand von 22,9 ± 126,9 min auf 6,0 ± 49,5 min.

Zusatzdiagnostik ohne zugehörige klinische Untersuchung erhielten im Geltungszeitraum der 3. FS 215 Patienten (3,5 %; 277 Untersuchungen). Dabei handelte es sich bei 165 Patienten um den ersten Untersuchungsschritt. Nach Inkrafttreten der 4. FS waren hiervon noch 37 bzw. 6 Patienten (0,6 % bzw. 0,1 %) betroffen. In späteren Untersuchungsgängen betraf dies nahezu ausschließlich Patienten, bei denen vormals die klinischen Ausfallzeichen dokumentiert wurden und ZD negativ oder nicht verwertbar war. Bei 2091 Patienten (34,3 %) im Geltungszeitraum der 3. FS erfolgte in mindestens einem Untersuchungsgang ZD vor Abschluss beider klinischer Untersuchungen. Nach der Novelle waren dies nur noch 126 Patienten (2,3 %).

Das Intervall zwischen klinischer Untersuchung und ZD innerhalb eines Untersuchungsganges betrug im Geltungszeitraum der 3. FS 2,2 ± 4,9 h, bei 6,2 % der Untersuchungen mehr als 6 h. Nach Inkrafttreten der 4. FS waren dies 3,1 ± 6,5 h und 7,9 %. Kürzere Abstände fanden sich bei EEG (2,4 ± 5,3 h), TCD (1,8 ± 4,4 h) und evozierten Potenzialen (3,1 ± 6,9 h), längere bei CTA (3,9 ± 7,9 h) und Szintigraphie (4,8 ± 7,8 h). Der mittlere Abstand war im BD bei allen ZD-Modalitäten um ca. 1–2 h geringer.

**Figure 3:**
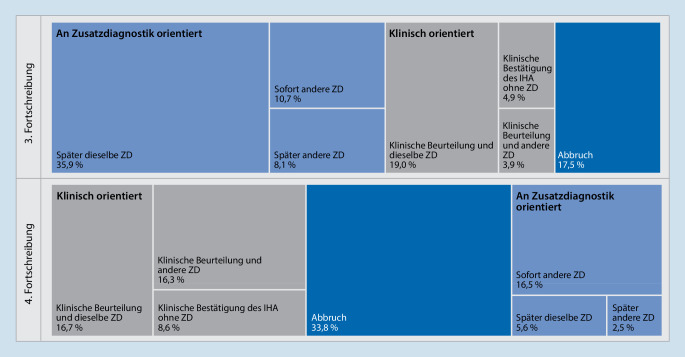

Das Vorgehen im Fall negativer ZD änderte sich deutlich (Abb. 3). Während vor der 4. FS die Diagnostik überwiegend mit weiterer ZD fortgesetzt wurde, erfolgte nun zuerst meist ein erneuter klinischer Untersuchungsgang. Wesentlich häufiger als zuvor wurde die Diagnostik abgebrochen. In beiden Zeiträumen wurde nur selten unmittelbar eine andere ZD angewendet oder nach vorgeschriebener Beobachtungszeit der klinische Irreversibilitätsnachweis angestrebt.

## Diskussion

Mit 11.503 Patienten liegt ein umfassender Überblick über die Praxis der IHA-Diagnostik in Deutschland 4 Jahre vor und nach Inkrafttreten der 4. FS vor. Einschränkungen resultieren aus der retrospektiven Auswertung anonymisierter Routinedaten. Wichtige Informationen wie der Abstand zwischen Eintritt des Hirnfunktionsausfalls und Beginn der Diagnostik, die Lokalisation der primären Hirnschädigungen und Sedativaspiegel waren nicht verfügbar, und die Gründe für das konkrete Vorgehen bleiben offen.

Zum IHA führen überwiegend primäre Hirnschädigungen [6]. Über den Beobachtungszeitraum nahm der Anteil sekundärer Hirnschädigungen zu, am deutlichsten in den C‑Krankenhäusern. Dagegen sank der Anteil von SHT, spontanen ICB und Hirninfarkten. Laut amtlicher Statistik nahmen tödliche Verkehrsunfälle zwischen 2010 und 2020 um etwa ein Drittel ab [16], nicht hingegen tödliche ICB und Hirninfarkte [17]. Zu vermuten sind daher veränderte Vorgehensweisen, die sich auf den Einsatz der Intensivtherapie sowie Eintritt, Feststellung und Meldung des IHA auswirken [7, 15].

Seit der 4. FS muss mindestens einer der klinischen Untersucher Facharzt für Neurologie oder Neurochirurgie oder Neuropädiater sein. Schon zuvor waren diese Gruppen mehrheitlich an der Diagnostik beteiligt. Nach Inkrafttreten wurde die Vorgabe bei allen Untersuchungen umgesetzt, die in die Diagnose des IHA einflossen. Weitere Untersuchungsgänge ohne Neuromediziner hatten vermutlich orientierenden Charakter. Dennoch wurde IHA-Diagnostik in C‑Krankenhäusern nun seltener abgeschlossen. Dies könnte dafür sprechen, dass neuromedizinische Expertise nicht jederzeit verfügbar war.

Einen Lösungsansatz bieten IHA-Konsildienste [4, 7]. Nach der Novelle nahmen in den meisten Regionen externe Konsile zu, besonders in C‑Krankenhäusern. Sie erfolgten oft im BD. Trotz hierdurch denkbarer Verzögerung wäre anderenfalls vermutlich ein noch stärkerer Rückgang der IHA-Diagnostik aufgetreten.

Die Verfügbarkeit qualifizierter Untersucher wurde vom Gesetzgeber als limitierender Faktor erkannt. Mit Änderung vom 11.07.2021 ist laut § 9a TPG die DSO als koordinierende Stelle aufgerufen, flächendeckend einen regional jederzeit verfügbaren Rufbereitschaftsdienst für die IHA-Diagnostik zu organisieren, an dem sich an Krankenhäusern tätige sowie niedergelassene Neurochirurgen und Neurologen auf Anfrage beteiligen. Bei der Umsetzung bestehen aus Sicht der Autoren – ungeachtet einer laut Gesetz angemessenen Vergütung – ungeklärte Fragen, darunter nach den Möglichkeiten der koordinierenden Stelle bei fehlender Teilnahmebereitschaft. Fraglich scheint auch, wie die Auswirkungen auf die Leistungsfähigkeit der Krankenhäuser kompensiert würden, wenn diese hochqualifizierte Fachärzte für eine zusätzliche BD-Schiene mit durchgehender Verfügbarkeit abordnen sollen. Aktuell bestehen regional stark heterogene Lösungsansätze für IHA-Konsile. Während feste Konsiliarteams einen verlässlichen Dienstplan vorhalten, stellen eigene Konsiliarbeziehungen der Entnahmekrankenhäuser oder das sog. „Lasso-Prinzip“, nach dem die DSO fallbezogen auf die Suche nach verfügbaren Konsiliaren geht, weniger stabile Lösungen dar. Eine stärkere Beteiligung von Fachärzten für Neurochirurgie, die im ausgewerteten Zeitraum lediglich 6,4 % der externen Konsile durchführten, scheint wünschenswert.

Trotz der nun verpflichtenden Einbindung von Pädiatern zeigte sich keine Abnahme der Untersuchungszahlen in den pädiatrischen Altersgruppen. Fast nie wurden Pädiater als externe Konsiliare tätig. Vermutlich erfolgte die Diagnostik in Einrichtungen, die die höheren Anforderungen bereits erfüllten, sodass die 4. FS den Status quo festschrieb. Ob Verlegungen im Vorfeld erfolgten, ist unbekannt.

Ungeachtet der Ergebnisse dürften Herausforderungen bei der Qualifizierung zukünftiger Untersucher nicht nur in der Pädiatrie weiterbestehen [12]. Mit Blick auf die sinkende und ungleichmäßig verteilte Zahl der IHA-Untersuchungen kann der Erwerb praktischer Fähigkeiten innerhalb der klinischen Facharztweiterbildung nicht mehr überall garantiert werden. Somit kommt externen Fortbildungen, Praxisworkshops und Simulationskursen steigende Bedeutung zu, die bereits von einigen Landesärztekammern und auf Fachkongressen wie dem der Deutschen Interdisziplinären Vereinigung für Intensiv- und Notfallmedizin (DIVI) angeboten werden.

Mehr als 80 % der Patienten erhielten ZD. Sie ist obligatorisch vor dem 3. Lebensjahr sowie bei primärer infratentorieller Hirnschädigung. Zudem kann sie anstelle von klinischer Verlaufsbeurteilung zum Irreversibilitätsnachweis eingesetzt werden, wenn eine primäre supratentorielle oder sekundäre Hirnschädigung ab Beginn des 3. Lebensjahres vorliegt. ZD erfolgt meist in dieser fakultativen Indikation und reduziert das Risiko des finalen Kreislaufstillstandes vor Abschluss der Diagnostik [5].

Durch Nachweis des zerebralen Perfusionsstillstandes kann zudem bei eingeschränkter klinischer Beurteilbarkeit die Vollständigkeit des IHA belegt werden. Allerdings sind Szintigraphie und TCD nicht umfassend verfügbar, und die Aussagekraft des TCD kann z. B. nach offenem SHT, Kraniektomie oder bei fehlendem Schallfenster eingeschränkt sein [18]. Seit der 4. FS ist die CTA als ZD für Erwachsene zugelassen. Sie ist grundsätzlich an jedem Krankenhaus verfügbar. Sie avancierte unmittelbar zur zweithäufigsten Form der ZD und trug zum Anstieg von IHA-Diagnostik mit ZD bei. In ca. 90 % wies sie den Perfusionsstillstand nach. Unerwartet war der sehr niedrige Anteil nicht auswertbarer Untersuchungen; hier besteht aus Sicht der Autoren der Verdacht einer nicht vollständigen Meldung.

Das seit der 4. FS vorgegebene CTA-Protokoll (Infobox) wurde bei 71 Patienten mit klinischem Hirntodsyndrom evaluiert [19]. Die Validität war mit 94 % ähnlich wie bei TCD (92 %). Vorliegend stellte die parallele Untersuchung mit CTA und TCD eine Rarität dar. In keinem Fall wurde ein zuvor festgestellter Perfusionsstillstand nicht bestätigt. Negative Befunde waren im TCD häufiger als in der CTA. Falsch-negative TCD-Befunde sind möglich, wenn die infraophthalmische A. carotis interna durch Versorgung der A. ophthalmica perfundiert bleibt. In der CTA ist dieser Befund mit dem zerebralen Perfusionsstillstand vereinbar [2, 18]. Negative CTA waren häufiger, je länger die klinischen Ausfallzeichen bestanden. Erneute CTA oder TCD blieben hier oft negativ, während Szintigraphie und EEG meist positiv ausfielen. Somit scheint bei negativer CTA primär ein Wechsel des Verfahrens ratsam. Eine erneute CTA oder ein TCD könnten aussichtsreicher sein, wenn das Hirntodsyndrom weniger als 36 h bestand [11].

Im Geltungszeitraum der 3. FS waren längere Zeitabstände innerhalb eines Untersuchungsganges sowie das Voranstellen von ZD nicht unüblich. Die 4. FS verdeutlichte, dass die Sequenz aus Klärung der Voraussetzungen, Feststellung der Ausfallzeichen durch zwei klinische Untersucher und Nachweis der Irreversibilität durch ZD und/oder einen weiteren klinischen Untersuchungsgang nach definierter Beobachtungszeit einzuhalten ist. Diese Anforderung wurde seit Inkrafttreten der 4. FS umgesetzt, wobei ZD auch weiterhin oft mit mehrstündiger Latenz erfolgte. Kürzere Wartezeiten im BD könnten auf kapazitäre Konflikte mit der regulären Patientenversorgung hinweisen.

In beiden Zeiträumen wurde bei negativer ZD nur nachrangig der rein klinische Irreversibilitätsnachweis angestrebt oder unmittelbar weitere ZD im selben Untersuchungsgang angeschlossen, wie dies in der 4. und 5. FS dargestellt ist [2, 3]. Vor Inkrafttreten der 4. FS wurde am häufigsten dieselbe ZD wiederholt, bevor ggf. erneute klinische Untersuchungen folgten. Nach Inkrafttreten geschah dies nur noch selten. Hingegen wurde nun bei 33,8 % die Diagnostik abgebrochen. Beides könnte dafür sprechen, dass einige Ärzte negative ZD als unvereinbar mit dem IHA ansehen. Negative ZD hat allerdings bei vollständig beurteilbaren Patienten, bei denen eine plausible und unumkehrbare Ursache zum vollständigen klinischen Hirnfunktionsausfall geführt hat, keinen prädiktiven Wert hinsichtlich der Reversibilität [20]. Auch vorliegend wurde kein derartiger Fall erfasst. Weitere Gründe für den häufigeren Therapieabbruch können eine zunehmende Rolle therapiebegrenzender Patientenverfügungen und palliativ ausgerichtete Therapiezieländerungen sein [7, 8]. Wünschenswert scheint weitere intensive Diskussion in der Öffentlichkeit und innerhalb der Fachgesellschaften über die möglichen Zielkonflikte, wenn gleichzeitig die Bereitschaft zur Organspende und der Wunsch nach Therapiebegrenzung bestehen [9, 14].

Die Autoren der Richtlinie betonen die prinzipielle Unabhängigkeit der IHA-Feststellung von der Frage einer Organ- oder Gewebespende. Zugleich formulieren sie aber das Ziel, Unsicherheiten und Ängsten entgegenzutreten und das Vertrauen in die sichere Todesfeststellung zu stärken [2]. Im Vergleich der 3. und 4. FS nahmen die gemeldeten Untersuchungen um ca. 11 % ab. Schon vor 2015 führten aufgedeckte Manipulationen in Transplantationszentren zum Rückgang der Organspendebereitschaft [10]. Bereits davor sank die Zahl der Organspenden [15]. Es kann somit zwar nicht auf eine negative Auswirkung der 4. FS geschlossen werden. Andererseits konnte sie offenbar ebenso wenig wie zwischenzeitliche Änderungen am TPG, die auf strukturelle Verbesserungen und Anreize für die Entnahmekrankenhäuser sowie eine verbesserte Aufklärung der Bevölkerung zielten, das weitere Absinken der Zahl realisierter Organspenden verhindern [13]. Auch nach Inkrafttreten der jüngsten 5. FS bleibt ein Spannungsfeld bestehen zwischen dem Anspruch, das Vertrauen in eine sichere Todesfeststellung durch präzise Vorgaben weiter zu stärken, und den Problemen ihrer praktischen Umsetzung, die sich potenziell limitierend auf die Organspende auswirken können [7].

### Schlussfolgerung

Die seit der 4. FS geltenden Anforderungen an die Untersucher und den Ablauf der Diagnostik wurden richtlinienkonform umgesetzt. Die zwingende Mitwirkung von Neuromedizinern erhöht besonders in C‑Krankenhäusern den Bedarf an externen Konsilen. Dagegen erfolgt pädiatrische IHA-Diagnostik in Einrichtungen, die die neuen Anforderungen aus eigener Kraft umsetzen können. Die CTA wird als neues ZD-Verfahren flächendeckend erfolgreich eingesetzt. Der Zeitbedarf für die Diagnostik stieg um ca. 20 %.

#### Infobox CT-Angiographie-Protokoll zur Feststellung des zerebralen Perfusionsstillstandes [2, 3]

- Zulässig nur bei Erwachsenen nach Protokollierung der klinischen Ausfallsymptome und bei einem arteriellen Mitteldruck über 60 mm Hg
- Nativscan: Gantryeinstellung parallel zur Orbitomeatallinie, Spiralscan von Schädelbasis bis Vertex mit 120 kV, 170 mA. Rekonstruierte axiale Aufnahmen in 5‑mm-Schichtdicke
- Druckinfusion von 65 ml hochkonzentriertem nichtionischem Kontrastmittel sowie 30 ml NaCl 0,9 % als Nachlauf; Förderrate 3,5 ml/s über peripheren oder zentralen Venenkatheter
- CT-Angiographie: automatischer Start über Bolustracking 5 s nach Erreichen von 150 Hounsfield-Einheiten in der A. carotis communis; Spiralscan von Halswirbelkörper (HWK) 6 bis Vertex mit 120 kV, 200 mA, Tischvorschub 4 cm/s (alternativ: simultaner Volumenscan von HWK 6 bis Vertex 15 s nach Ankunft des Bolus); axiale Rekonstruktion in 2 mm Schichtdicke
- Auswertung: Der zerebrale Zirkulationsstillstand ist nachgewiesen bei folgender Befundlage:
  - *Keine* Kontrastierung der A. basilaris, der M1-Segmente beider Aa. cerebri mediae, der A1-Abschnitte beider Aa. cerebri anteriores sowie der P1-Abschnitte beider Aa. cerebri posteriores *und*
  - *nachgewiesene* Kontrastierung beider Aa. carotides communes, Aa. carotides externae und ihrer Äste, insbesondere der Aa. temporales superficiales
  - Proximale Abschnitte der intraduralen Aa. carotides internae, der V4-Segmente oder der Aa. inferiores posteriores cerebelli (PICA) können durch „stasis filling“ auch bei zerebralem Zirkulationsstillstand kontrastiert sein
- Befundbeurteilung durch einen Neuroradiologen oder einen Facharzt für Radiologie mit mehrjähriger Erfahrung in der neuroradiologischen Diagnostik
- Eine wesentliche Fehlerquelle ist die fehlerhafte Platzierung des Messvolumens für das Bolustracking außerhalb der A. carotis communis. Eine unmittelbare Wiederholung ist nach bereits erfolgter Kontrastmittelgabe nicht möglich

## Fazit für die Praxis

- Auch in der 5. Fortschreibung der Richtlinie müssen bei der Feststellung des irreversiblen Hirnfunktionsausfalls (IHA) die Vorgaben zur Qualifikation der klinischen Untersucher und zur Abfolge der Untersuchungsschritte beachtet werden.
- Die Gleichwertigkeit von apparativer Zusatzdiagnostik (ZD) und erneuter klinischer Beurteilung nach definierter Wartezeit zum Irreversibilitätsnachweis bei vollständig beurteilbaren Patienten mit primärer supratentorieller oder sekundärer Hirnschädigung ab Beginn des 3. Lebensjahres bleibt zentraler Bestandteil des gültigen IHA-Konzepts.
- Apparative ZD kann zur Bestätigung des IHA nur herangezogen werden, wenn sie im direkten Zusammenhang nach Feststellung der klinischen Ausfallzeichen erfolgt.
- Im Fall negativer ZD kann eine andere ZD-Modalität unmittelbar angeschlossen werden, um die Irreversibilität des IHA zu belegen.
- Die CT-Angiographie ist eine aussagekräftige und überall verfügbare Option zur ZD. Sie kann die IHA-Feststellung maßgeblich unterstützen. Die von der Routine abweichende Methodik sollte bereits im Vorfeld etabliert werden.
